# A Study on Lower Limb Asymmetries in Parkinson’s Disease during Gait Assessed through Kinematic-Derived Parameters

**DOI:** 10.3390/bioengineering9030120

**Published:** 2022-03-16

**Authors:** Federico Arippa, Bruno Leban, Marco Monticone, Giovanni Cossu, Carlo Casula, Massimiliano Pau

**Affiliations:** 1 Department of Mechanical, Chemical and Materials Engineering, University of Cagliari, 09123 Cagliari, Italy; federico.arippa@unica.it (F.A.); bruno.leban@unica.it (B.L.); 2 Neurorehabilitation Unit, Department of Neuroscience and Rehabilitation, ARNAS “G. Brotzu”, 09134 Cagliari, Italy; marco.monticone@unica.it; 3 Department of Medical Sciences and Public Health, University of Cagliari, 09042 Monserrato, Italy; 4 Neurophysiology and Movement Disorders Unit, Department of Neurology, ARNAS “G. Brotzu”, 09134 Cagliari, Italy; giovannicossu1@gmail.com; 5 Physical Medicine and Rehabilitation Unit, ARNAS “G. Brotzu”, 09134 Cagliari, Italy; carlocasula@aob.it

**Keywords:** Parkinson’s disease, gait, kinematics, symmetry

## Abstract

Unilaterality of motor symptoms is a distinctive feature of Parkinson’s Disease (PD) and represents an important co-factor involved in motor deficits and limitations of functional abilities including postural instability and asymmetrical gait. In recent times, an increasing number of studies focused on the characterization of such alterations, which have been associated with increased metabolic cost and risk of falls and may severely compromise their quality of life. Although a large number of studies investigated the gait alterations in people with PD (pwPD), few focused on kinematic parameters and even less investigated interlimb asymmetry under a kinematic point of view. This retrospective study aimed to characterize such aspects in a cohort of 61 pwPD (aged 68.9 ± 9.3 years) and 47 unaffected individuals age- and sex-matched (66.0 ± 8.3 years), by means of computerized 3D gait analysis performed using an optical motion-capture system. The angular trends at hip, knee and ankle joints of pwPD during the gait cycle were extracted and compared with those of unaffected individuals on a point-by-point basis. Interlimb asymmetry was assessed using angle–angle diagrams (cyclograms); in particular, we analyzed area, orientation, trend symmetry and range offset. The results showed that pwPD are characterized by a modified gait pattern particularly at the terminal stance/early swing phase of the gait cycle. Significant alterations of interlimb coordination were detected at the ankle joint (cyclogram orientation and trend symmetry) and at the hip joint (range offset). Such findings might be useful in clinical routine to characterize asymmetry during gait and thus support physicians in the early diagnosis and in the evaluation of the disease progression.

## 1. Introduction

Cardinal motor symptoms such as bradykinesia, rest tremor and rigidity represent some of the most distinctive features of Parkinson’s Disease (PD) and originate from degeneration of nigral dopaminergic neurons [1]. Their presentation is typically asymmetric [2], as also confirmed by comparing data derived from imaging techniques of asymptomatic patients and those with mild-early symptoms [3,4,5], leading unilaterality to be considered as one of the main clinical features useful to discriminate PD from other Parkinsonisms [6,7]. It has been reported that unilaterality persists throughout the clinical course of the disease in many cases [8] as marked differences between motor functions of right and left sides remain evident for 30 years and up [6]. Such asymmetry also reflects on the Unified Parkinson’s Disease Rating Scale (UPDRS) score [9,10,11,12] and, usually, does not significantly change during the progression of the disease. This is confirmed by several studies which reported that worsening in the UPDRS motor scores (UPDRS-III part) progresses similarly on both sides [8].

In people with PD (pwPD) unilaterality causes postural instability and asymmetrical gait [13], which are associated with increased metabolic cost and risk of falls, and thus negatively affect the quality of life [14]. Given the pivotal role played by the locomotor abilities in several activities of daily living (ADL) and, generally speaking, on the quality of life of pwPD, in the last decade, researchers and clinicians highlighted the need to have available objective tools for timely detection of gait alterations (even when subtle), to characterize the disease progression and to monitor the effectiveness of pharmacologic and rehabilitative treatments. In such context, some studies attempted to investigate and quantify gait asymmetries in pwPD with particular focus on spatiotemporal parameters. Unfortunately, their findings are quite mixed: in fact, although some of them detected larger gait asymmetries in step length and step time parameters in pwPD with respect to unaffected individuals [14] as well as the existence of correlations between asymmetry of gait and disease severity [15], others did not [16,17]. However, all these studies share an important limitation, namely the fact that they focus their attention on discrete values of spatiotemporal parameters. Although such approach has the advantage to provide clinicians with an easily interpretable summary of the entire gait performance, discrete values may not always be sufficiently reflective of the complex alterations of lower limb movement connected to pathological gait conditions [18]. Moreover, as pointed out in a recent review [19], walking-related information in pwPD needs to be improved. Thus, methods that focus on the kinematics of the lower limb during the whole gait cycle may be able to better gather the complexity of locomotor alterations in pwPD.

To these authors’ knowledge, the existing study on lower limb kinematics of pwPD did not investigate on a point-by-point basis the difference in hip, knee and ankle joint angular trends with respect to unaffected individuals and, similarly, only few data exist in terms of interlimb symmetry. Since detection of asymmetry may support an early diagnosis of the disease, this additional information could be relevant for the clinician who first evaluates the pwPD, especially to support suitable recommendation of specific rehabilitation protocols, training programs, as well as healthier lifestyles. On the basis of such considerations, the main purpose of the present study was to extensively characterize lower limb kinematics in individuals diagnosed with idiopathic PD by providing summary indexes of gait quality, and symmetry parameters calculated from the angular trend associated with the entire gait cycle for each joint of interest.

## 2. Materials and Methods

### 2.1. Participants

Sixty-one pwPD admitted at the Neurologic Department of the ARNAS “G. Brotzu” General Hospital (Cagliari, Italy) underwent a 3D gait analysis at the Laboratory of Biomechanics and Industrial Ergonomics of the University of Cagliari (Cagliari, Italy). They were all diagnosed according to the UK Brain Bank criteria [20] by a trained expert neurologist (G.C.) and free from any other neurologic and orthopedic condition able to significantly influence gait or balance. Their motor functions were assessed using the motor section of UPDRS (UPDRS part III). The experimental trials were carried out in “ON” state (i.e., approx. 60 to 90 min after taking an appropriate oral dose of dispersible Levodopa). Forty-seven unaffected age- and sex-matched individuals recruited among the University and Hospital staff served as the control group (CG). 

The study was conducted according to the principles expressed in the World Medical Association Declaration of Helsinki. At the time of the tests, all participants signed a written informed consent form which included detailed information about the aims of the study and the experimental methodology.

### 2.2. Spatiotemporal and Kinematic Data Collection and Processing

Spatiotemporal and kinematics parameters of gait were assessed by means of an optical motion-capture system composed of 8 infrared cameras (Smart-D, BTS Bioengineering, Milan, Italy) running at a 120 Hz frequency. Before starting the experimental tests, anthropometric data (i.e., height, weight, anterior superior iliac spine distance, pelvis thickness, knee and ankle width, leg length) were acquired, and then 22 spherical reflective passive markers were placed on subjects’ skin in accordance with the protocol defined by Davis et al. [21]. All participants were instructed to walk at a self-selected speed as naturally as possible along a 10 m walkway, while the 3D marker’s trajectories were acquired by the cameras. The test was considered valid if at least 6 trials were correctly recorded, in order to have available an adequate number of gait cycles for the subsequent processing. Suitable periods of rest between consecutive trials were allowed on request. At the end of the tests, raw data were processed with a dedicated software (Smart Analyzer, BTS Bioengineering, Milan, Italy) to calculate:

- Spatiotemporal gait parameters (i.e., gait speed, cadence, step length, step width, stance, swing and double support phase duration);
- Kinematic parameters (pelvic tilt, rotation and obliquity; hip flexion–extension, adduction–abduction and rotation; knee flexion–extension, ankle dorsi–plantarflexion, and foot progression). From these parameters, additional indexes on gait deviation from normality were obtained, namely the Gait Variable Scores (GVS) and Gait Profile Score (GPS) [22];
- Dynamic range of motion (ROM) for hip and knee flexion–extension and ankle dorsi–plantarflexion. Values were obtained as the difference between the maximum and minimum angle value recorded during the gait cycle;
- Sagittal kinematics of hip, knee and ankle (i.e., hip and knee flexion–extension and ankle dorsi–plantarflexion angles during the gait cycle) which were also employed to calculate the interlimb symmetry parameters as described later in detail.

Additionally, asymmetry between right and left limb in terms of spatiotemporal parameters was quantified on the basis of the Symmetry Index (SI) proposed by Robinson et al. [23]:

$$SI=ABS\left(2\times \frac{V_{R}-V_{L}}{V_{R}+V_{L}}\times 100\right)$$

where *V_R_* and *V_L_* represent the values of the gait variable (in our case stance, swing, double support duration phases and step length) for the right and left limb. Such a method, originally proposed for the evaluation of symmetry in ground reaction force during gait, is one of the most used indexes in gait symmetry studies, and has been also modified so as to include spatiotemporal, kinematic parameters, as well as muscle activity data [18].

### 2.3. Inter-Limb Symmetry Quantification by Means of Waveform-Based Method

**Figure 1:**
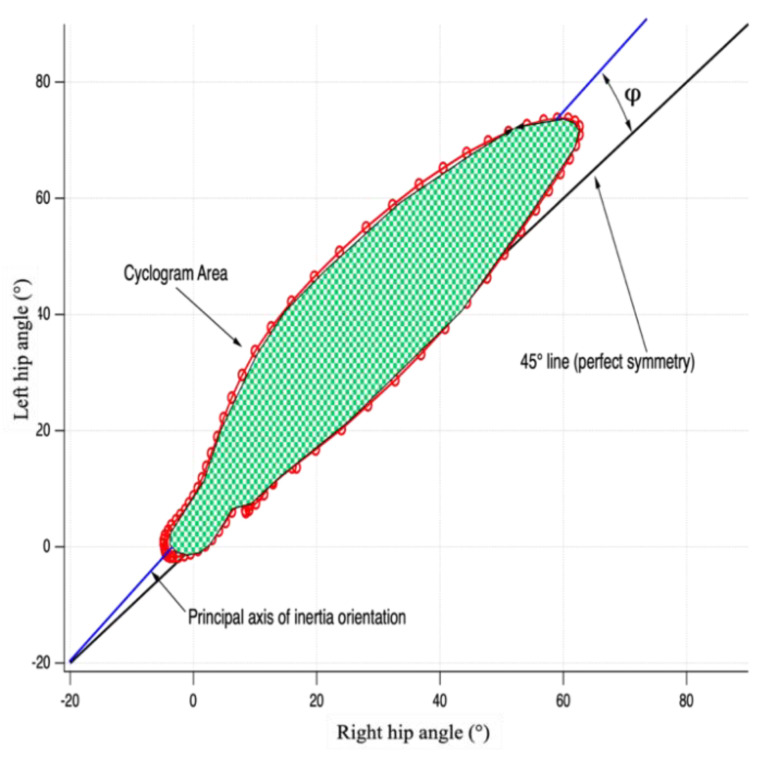
Graphic representation of a cyclogram and its main features considered for the present study.

Bilateral cyclograms were calculated using a dedicated software developed under Matlab environment basing on the procedure proposed by Goswami [24] which requires right and left limb angles at hip, knee and ankle joints during the gait cycle to build left–right-angle diagrams from which the following symmetry parameters were calculated (Figure 1):

- Cyclogram area (degrees^2^): area enclosed by the curve obtained from the left–right angle diagram [25]. A hypothetical symmetrical gait would lead left and right joints to assume the same angular position during the gait cycle. In this way, cyclogram points would lie on a 45° line in the diagram with a null area;
- Cyclogram orientation (degrees): this parameter is expressed as the absolute value of the angular difference φ between the perfect symmetry line (45° line) and the orientation of the principal axis of inertia [24,26], which is the direction of the eigenvector of the inertial matrix for the cyclogram points in the x–y (left vs. right joint angle) reference system. Low φ angles indicate higher interlimb symmetry;
- Trend Symmetry (dimensionless): Calculated to assess the similarity of two waveforms (i.e., right and left leg angular trend across the gait cycles for each joint) by means of an eigenvector analysis [27]. Trend Symmetry index is obtained by dividing the variability about the eigenvector to the variability along the eigenvector and is not affected by a shift or magnitude differences in two considered waveforms. Low or null values indicate higher symmetry, and interlimb asymmetry results in high Trend Symmetry values;
- Range offset, a measure of the differences in operating range of each limb, is calculated as the absolute value of the difference between the average of the right-side waveform from the average of the left-side waveform [27]. In particular, this parameter indicates if one side operates in a wider flexion range than the opposite side; zero values indicate that both sides work within the same ROM.

### 2.4. Statistical Analysis

A statistical analysis was conducted to evaluate the effect of the disease on gait parameters of interest. In particular, all outcome measures were analyzed in order to investigate the existence of differences originated by the presence of PD. Separate one-way multivariate analysis of variance (MANOVA) were performed, considering group (PD/CG) as the independent variable while the spatiotemporal parameters, SI, GPS and GVSs, ROM were set as dependent variables. In the case of spatiotemporal parameters, they were separated into two groups:

1. Gait speed, cadence and step width, for which both limbs are involved;
2. Stance, swing, double support phases and step length, where only one limb is involved.

To investigate symmetry in joint kinematics, and to assess in which periods of the gait cycle significant differences associated with PD occurred, the angle-cycle curves for PD vs. CG were compared on a point-by-point basis using a one-way ANOVA, setting the group as independent variable. This analysis was performed for each of the 3 joints of interest [28].

Finally, the existence of significant differences in inter-limb symmetry due to PD was also investigated by means of a MANOVA, with group (PD or CG) as the independent variable and the 4 symmetry parameters (cyclogram area and orientation, trend symmetry and range offset) as dependent variables. 

A preliminary analysis was performed to exclude the existence of significant differences in the investigated parameters between left and right limbs. Since no significant differences were found, the mean value of each parameter calculated across the two limbs was considered for each participant. 

In all above cases, the level of significance was set at *p* = 0.05 and the effect sizes were assessed using the eta-squared (η^2^) coefficient. Univariate analysis of variance (ANOVA) was carried out, when necessary, as a post hoc test by reducing the level of significance according to the Bonferroni correction. All analyses were performed using the SPSS version 26 software (IBM SPSS Statistics, Armonk, New York, NY, USA).

## 3. Results

Demographic, anthropometric and clinical characteristics of the participants are reported in Table 1, while the results of the comparison for spatial–temporal parameters, GPS, GVSs, dynamic ROM and symmetry indexes between pwPD and the CG are reported in Table 2, Table 3, Table 4 and Table 5.

### 3.1. Spatiotemporal Parameters of Gait

Significant effect originated by the presence of PD on spatiotemporal parameters of gait was detected by MANOVA for both, single limb and double limb related parameters [F(4,106) = 4.286, *p* = 0.003, Wilks λ = 0. 857, η^2^ = 0.143] and [F(3,106) = 4.378, *p* = 0.006, Wilks λ = 0.888, η^2^ = 0.112], respectively. In particular, the follow-up ANOVA (Table 2) indicated that pwPD exhibit reduced speed, step length and swing phase duration and increased double support phase duration when compared to unaffected individuals.

**Table 2:** Spatiotemporal parameters of gait. Stance, swing and double support phases are expressed as percentage of the gait cycle duration. Values are expressed as mean ± SD.

	Control Group	PD Group
Speed (m/s)	1.18 ± 0.22	1.06 ± 0.26 **
Cadence (steps/min)	112.32 ± 10.24	111.49 ± 12.99
Step Length (m)	0.63 ± 0.08	0.55 ± 0.11 **
Step Width (m)	0.20 ± 0.02	0.19 ± 0.04
Stance Phase (% of the gait cycle)	59.96 ± 1.65	60.77 ± 2.62
Swing Phase (% of the gait cycle)	40.06 ± 1.65	38.67 ± 2.47 **
Double Support Phase (% of the gait cycle)	20.07 ± 3.29	22.60 ± 4.73 **

The symbol ** denotes a significant difference with respect to the Control Group (in all cases *p* < 0.01).

Significant effect of PD for spatiotemporal SI (Table 3) was detected by MANOVA analysis [F(4,106) = 5.574, *p* = 0.000, Wilks λ = 0. 822, η^2^ = 0.178]. In particular, SI values were significantly higher in PD subjects for double support and step length parameters (*p* = 0.017 and *p* = 0.001, respectively).

**Table 3:** SI parameters of gait. Stance, swing and double support phases are expressed as percentage of the gait cycle duration. Values are expressed as mean ± SD.

Symmetry Index	Control Group	PD Group
Step Length	2.90 ± 1.92	4.90 ± 3.52 **
Stance Phase Duration	1.66 ± 1.20	2.39 ± 2.94
Swing Phase Duration	2.45 ± 1.79	3.62 ± 4.04
Double Support Phase Duration	7.90 ± 6.29	14.22 ± 17.03 *

The symbols * and ** denote a significant difference with respect to the Control Group (* *p* < 0.05, ** *p* < 0.01).

### 3.2. Gait Kinematics, GPS and GVS

The statistical analysis revealed a significant main effect of group on GPS and GVS indexes [F(10,104) = 2.622, *p* = 0.007, Wilks λ = 0.784, η^2^ = 0.216]. The follow-up ANOVA showed that pwPD exhibit increased GPS in comparison to CG (*p* < 0.01) and increased GVS for pelvic obliquity and rotation (*p* < 0.05 and *p* < 0.001, respectively) and knee flex-extension (*p* < 0.01). Mean values along with standard deviations for each group are reported in Table 4.

**Table 4:** GPS and GVS indexes (in degrees). Values are expressed as mean ± SD.

	Control Group	PD Group
GPS	6.60 ± 1.35	7.37 ± 1.31 **
Pelvic Obliquity GVS	2.23 ± 0.88	2.60 ± 0.95 *
Pelvic Tilt GVS	5.45 ± 3.14	6.04 ± 3.55
Pelvic Rotation GVS	3.35 ± 1.02	4.18 ± 1.34 **
Hip Abduction–Adduction GVS	3.69 ± 1.29	3.94 ± 1.29
Hip Flexion–Extension GVS	7.96 ± 3.35	8.54 ± 4.14
Hip Rotation GVS	7.83 ± 3.23	8.86 ± 3.14
Knee Flexion–Extension GVS	7.61 ± 2.51	8.98 ± 2.69 **
Ankle Dorsi–plantarflexion GVS	5.84 ± 1.99	6.34 ± 2.21
Foot Progression GVS	7.94 ± 2.60	8.47 ± 3.64

The symbols * and ** denote a significant difference with respect to the Control Group (* *p* < 0.05, ** *p* < 0.01).

### 3.3. Dynamic ROM

The statistical analysis detected the existence of significant main effect of group [F(3,105) = 5.015, *p* = 0.003, Wilks λ = 0.873, η^2^ = 0.127] on dynamic ROM during gait (Table 5). In particular, the follow-up ANOVA indicated that pwPD are characterized by significantly reduced ROM at hip and knee joints with respect to CG (*p* < 0.001 and *p* < 0.01, respectively).

**Table 5:** Dynamic range of motion during gait (in degrees). Values are expressed as mean ± SD.

Joint	Control Group	PD Group
Hip ROM	46.52 ± 6.11	42.02 ± 5.89 **
Knee ROM	58.80 ± 4.67	55.69 ± 5.53 **
Ankle ROM	26.47 ± 4.94	24.93 ± 4.98

The symbol ** denotes a significant difference with respect to the control group (*p* < 0.01).

### 3.4. Point-by-Point Analysis of Kinematic Curves

**Figure 2:**
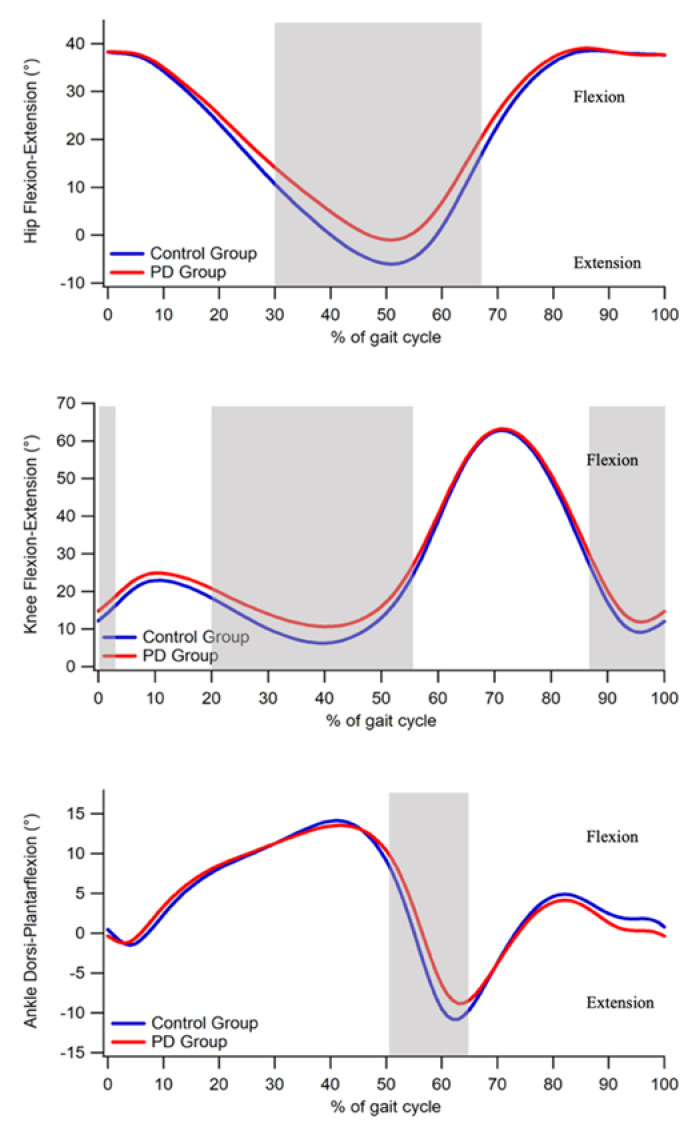
Gait kinematics in the sagittal plane. From top to bottom: hip flexion–extension, knee flexion–extension and ankle dorsi–plantar-flexion angles during gait cycle. Gray-shaded areas denote the periods of the gait cycle in which a significant difference between groups existed (*p* < 0.05).

The analysis of hip, knee, and ankle kinematics in the sagittal plane (Figure 2) revealed the existence of:

- At the hip joint level, significant differences between pwPD and CG from 30 to 67% of the gait cycle;
- At the knee joint, between 1 and 3%, between 20 and 56% and between 87 and 100% of the gait cycle;
- At the ankle joint from 51 to 64% of the gait cycle.

### 3.5. Waveform-Based Symmetry Indexes

MANOVA detected a significant effect of group on symmetry indexes at hip and ankle joints (hip [F(4,103) = 4.825, *p* = 0.001, Wilks λ = 0.838, η^2^ = 0.162]; ankle [F(4,105) = 8.355, *p* < 0.001, Wilks λ = 0.753, η^2^ = 0.247]), while no main effect was found for the knee joint. In particular, the analysis showed that the range offset is significantly larger in pwPD at the hip joint level (*p* < 0.05), as well as for cyclogram orientation and trend symmetry at the ankle joint (*p* < 0.001 and *p* < 0.01, respectively) with respect to the unaffected individuals (Table 6).

**Figure 3:**
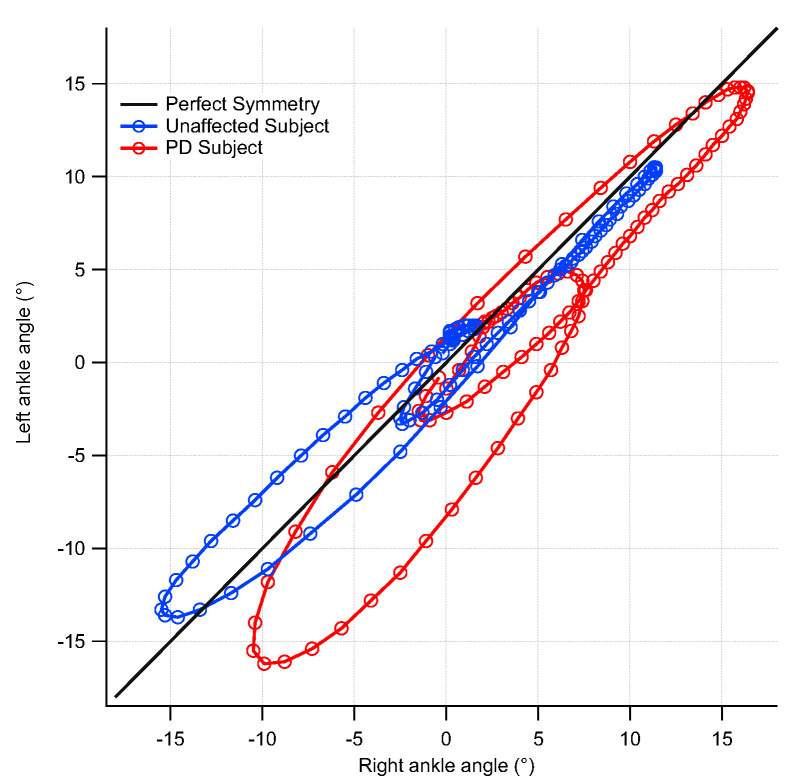
Comparison between cyclograms of an individual affected by PD and an unaffected individual. The diagram refers to the ankle joint.

Figure 3 shows an example of the different shapes and orientations of the cyclograms for PDs and unaffected individuals.

## 4. Discussion

The aim of the present study was to characterize alterations in gait kinematics among pwPD, with particular focus on the interlimb symmetry of hip, knee and ankle joints with respect to unaffected individuals. At first, it should be noted that the gait patterns of pwPD are substantially consistent with most previous studies in terms of spatiotemporal parameters, as they exhibit increased double support phase duration [29,30,31], reduced swing phase duration, step length and gait speed [32,33,34,35].

We found marked differences between pwPD and CG with regards to SI step length and SI double support, a result partly consistent with previous studies which also reported significantly larger asymmetries in pwPD for swing duration and step time other than step length and double support duration [13,14]. It is possible that such differences are due to the different conditions in which the participants were tested. In fact, while the quoted studies investigated gait with pwPD in OFF levodopa state, in our case we evaluated them while in the ON phase. It is thus likely that effect of medication somehow attenuated the gait alterations associated with the disease.

From a kinematic point of view, the analysis of the GVS parameters highlighted differences at the level of the hip and knee joints. In particular, consistent with what was reported by previous studies [29], pwPD were characterized by higher values in pelvic obliquity and rotation, and knee flexion–extension. Overall, such alterations originated a significantly higher value of the GPS with respect to unaffected individuals. Higher GVS scores for hip and knee flexion–extension may be due to reduced strength of muscles acting on these joints [36].

The point-by-point analysis of the sagittal kinematics showed significant alterations in pwPD for all the three joints with respect to the unaffected individuals, though of different magnitude. As regards the ankle joint, the main differences involved the terminal stance and swing phases of the gait cycle, where an increased dorsiflexion in pwPD was detected. This is consistent with previous studies [35,37], which indicated that reduced plantarflexion in PD group during the toe-off phase is caused by a decreased ankle power generation [37] due to a reduced amplitude of gastrocnemius activity [38]. Similarly, reduced knee extension was observed in the terminal stance phase and between 87 to 100% of the gait cycle. The author of [39] attributed this behavior to the reduced gait velocity and to the reduced ROM at the hip level, which was reflective of an increased rigidity among the pathological group.

One of the main aims of this study was the characterization of interlimb joint kinematics symmetry by means of a waveform-based method, which was previously employed with encouraging results for the characterization of asymmetries among people with osteoarthritis and multiple sclerosis [40,41]. Even though a certain asymmetry of gait kinematics exists even in a healthy population [42], neurodegenerative diseases such as PD are characterized by unilaterality [43], which is likely to result in larger asymmetries. The waveform-derived parameters employed in the present study showed that pwPD are characterized by a marked asymmetry at the ankle joint level, while slight differences exist for the hip joint level. In particular, at ankle joint cyclogram orientation and trend symmetry were twice and one and a half time higher, respectively, in PDs, who also showed higher values for range offset at the hip level. On the other hand, no significant differences were found for the knee joint. Despite some differences which were evidenced for the hip joint, our results somehow differ from Goswami [24] who also reported significant increases for cyclogram orientation values at the hip.

The observed asymmetries may be influenced by several factors. At first, as previously mentioned, in pwPD reduction in the microstructural integrity of the transcallosal fibers connecting homologous regions of the pre-supplementary motor and supplementary motor areas were observed, which were previously recognized as responsible for step length asymmetry [14]. A relevant role might also be played by the existence of asymmetries in muscular strength, which originate from right–left hemispheric asymmetry of the functional organization of basal ganglia [44]. Moreover, as previously reported, EMG activity of the gastrocnemius is reduced while walking [38], thus amplifying asymmetry at the ankle joint level, which reflects on alterations of cyclograms parameters.

Some limitations of the study should be acknowledged. First, the relative limited size of the sample here tested implies that generalization of the obtained results should be performed cautiously. Secondly, we had no information on the first or more affected limb in the PD group, and for this reason we used averages of left and right limbs when assessing differences in spatiotemporal parameters and ROM with respect to a healthy population. Information on the affected side may result in differences for these parameters for more vs. less affected side. Lastly, even though none of the participants exhibited freezing of gait during the tests, this phenomenon cannot be adequately captured by means of the setup employed here. Thus, specific tests should be planned to consider freezing of gait episodes in the analysis.

## 5. Conclusions

In this study we investigated the gait patterns of pwPD, focusing on lower limb joint kinematics, by comparing their joint angle curves with those of unaffected individuals and by calculating symmetry parameters derived from a waveform-based approach. The obtained results show that pwPD exhibit modified gait patterns characterized by severe modifications of the physiologic kinematic trend at the hip, knee and ankle level, especially during the terminal stance-early swing phase and final part of the gait cycle. The symmetry analysis revealed that the effect of the disease on interlimb coordination is present at the ankle joint and is moderate in the hip, while the knee joint appears relatively exempt from specific negative effects from this point of view. Such findings could be useful in clinical routine, since the quantitative information on asymmetry may represent an additional tool that helps clinicians to diagnose PD earlier and/or evaluate its development.

## Figures and Tables

**Table 1:** Demographic, anthropometric, and clinical characteristics of participants. Values are expressed as mean ± SD.

	Control Group (19 F, 28 M)	PD Group (24 F, 37 M)
Age (years)	66.0 ± 8.3	68.9 ± 9.3
Body mass (kg)	66.9 ± 11.1	67.1 ± 10.9
Height (cm)	164.7 ± 6.9	164.5 ± 7.8
Disease Duration (years)	-	7.7 ± 5.6
UPDRS III score	-	19.9 ± 9.3

**Table 6:** Comparison between symmetry indexes of PD and CG subjects. Values are expressed as mean ± SD.

	Cyclogram Parameter	Control Group	PD Group
	Area	116.57 ± 88.11	87.95 ± 72.18
Hip	Orientation *φ*	2.26 ± 2.36	1.92 ± 1.76
	Trend Symmetry	0.24 ± 0.21	0.29 ± 0.26
	Range Offset	2.27 ± 2.02	3.22 ± 2.08 *
	Area	268.76 ± 213.97	213.53 ± 156.65
Knee	Orientation *φ*	1.47 ± 1.40	1.62 ± 1.35
	Trend Symmetry	0.49 ± 0.42	0.48 ± 0.32
	Range Offset	4.52 ± 3.97	5.50 ± 3.22
	Area	62.52 ± 51.59	84.58 ± 63.71
Ankle	Orientation *φ*	1.99 ± 1.44	3.92 ± 2.80 *
	Trend Symmetry	1.54 ± 1.21	2.27 ± 1.48 *
	Range Offset	2.83 ± 2.05	3.57 ± 2.63

The symbol * denotes a significant difference with respect to the control group.

## Data Availability

Data will be made available on request.
